# Supplementary material for: The Role of Food Insecurity and Dietary Diversity on Recovery from Wasting among Hospitalized Children Aged 6–23 Months in Sub-Saharan Africa and South Asia
Source: Nutrients. 2022 Aug 24;14(17):3481. doi: 10.3390/nu14173481 (PMC9460249; doi:10.3390/nu14173481)
Supplement: Supplementary file 1 [file nutrients-14-03481-s001.zip › nutrients-1820385-supplementary.pdf]

Supplementary Materials

APPENDIX

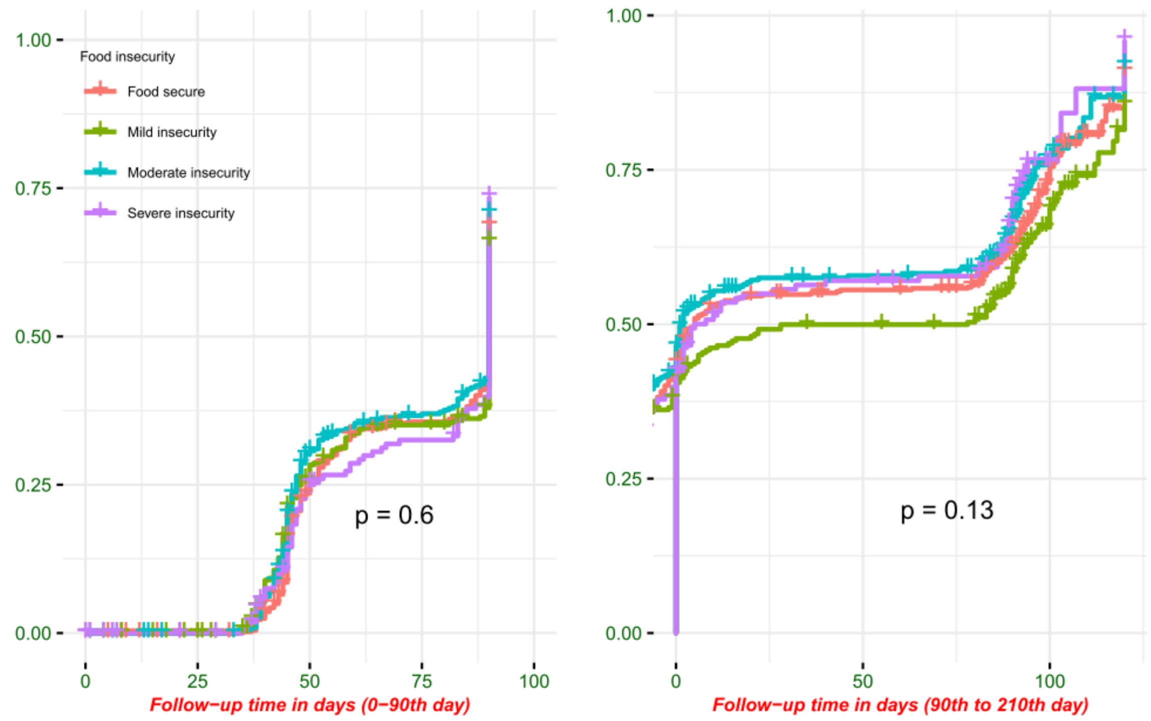

Figure S1. Plot for food insecurity over time

Models from The Sensitivity Analysis

Model 1: Individual missing follow-up MUAC considered as censored

Model 2: Complete case analysis

Model 3: Mixed-effects Cox proportional hazards model, accounting for random effect by study site

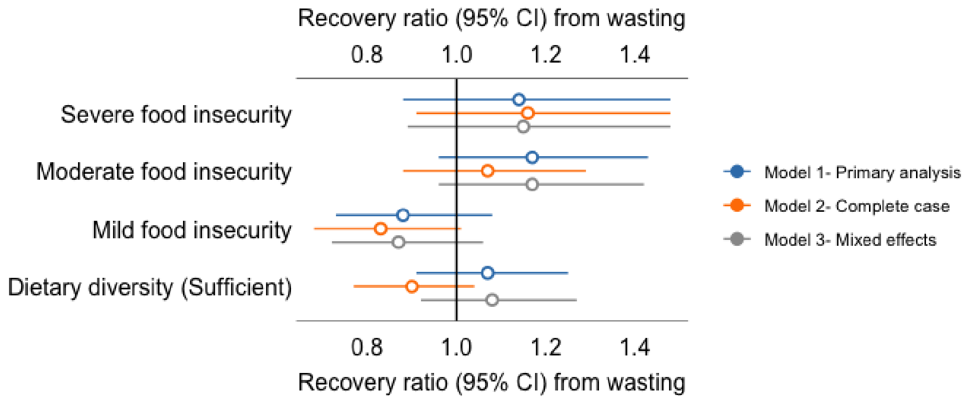

Figure S2: Sensitivity analysis for the effect of food insecurity and dietary diversity on recovery from acute malnutrition

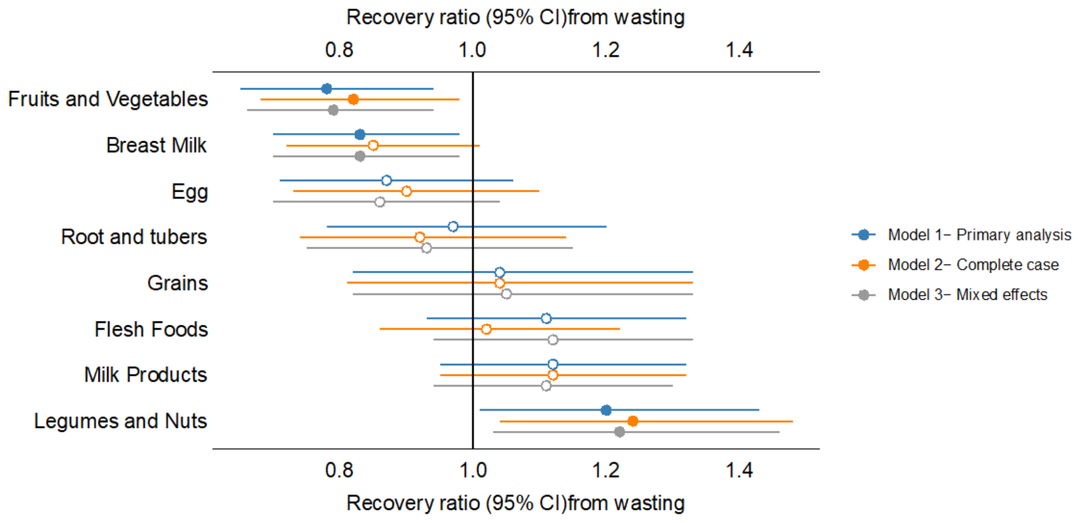

Figure S3: Sensitivity analysis for the effect of specific food groups on recovery from wasting

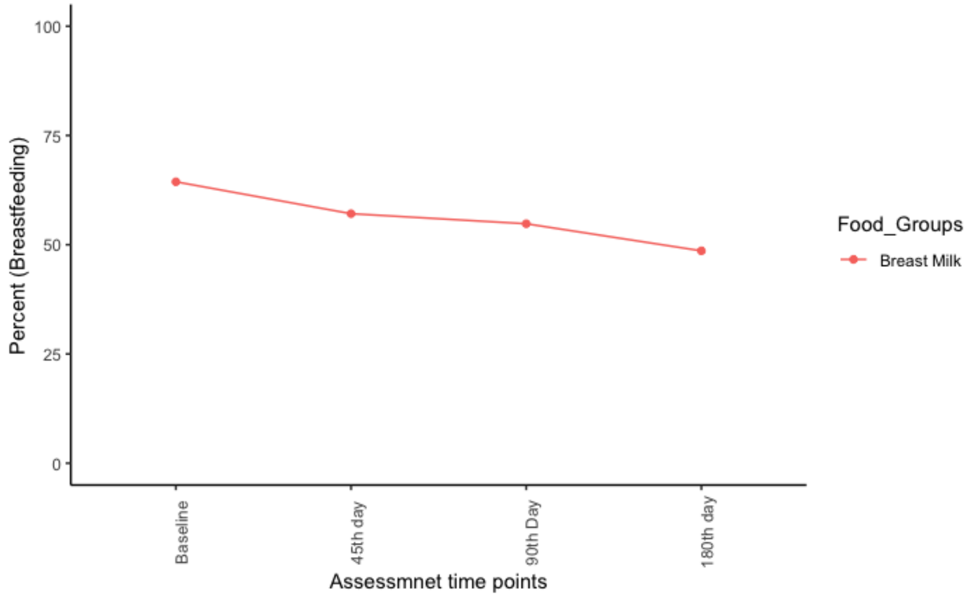

Figure S4. The trend of breastfeeding overtime

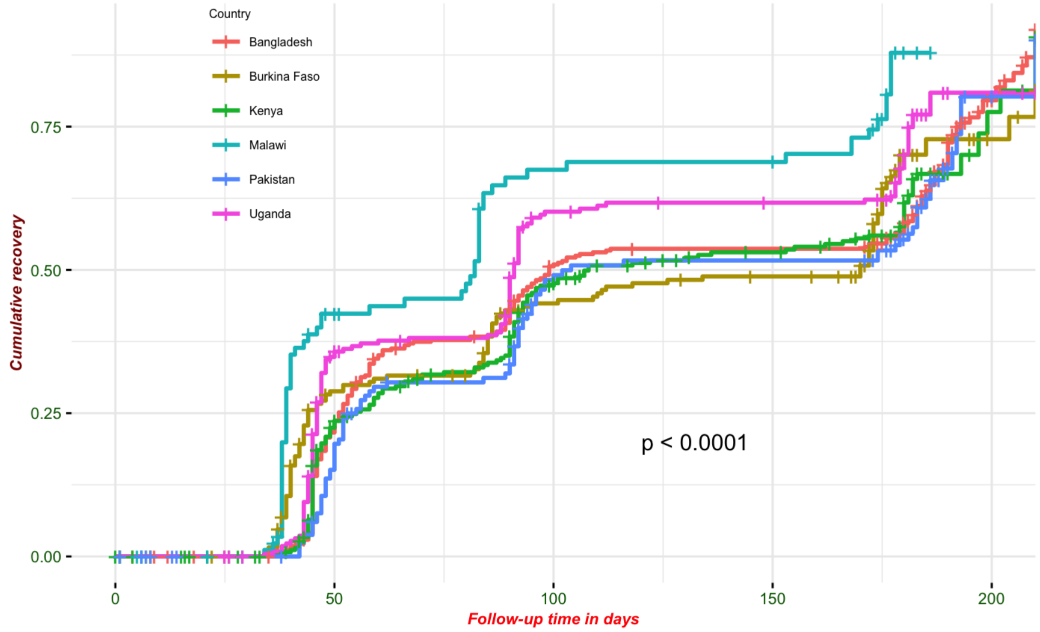

Figure S5. A Kaplan-Meier curve of recovery by country

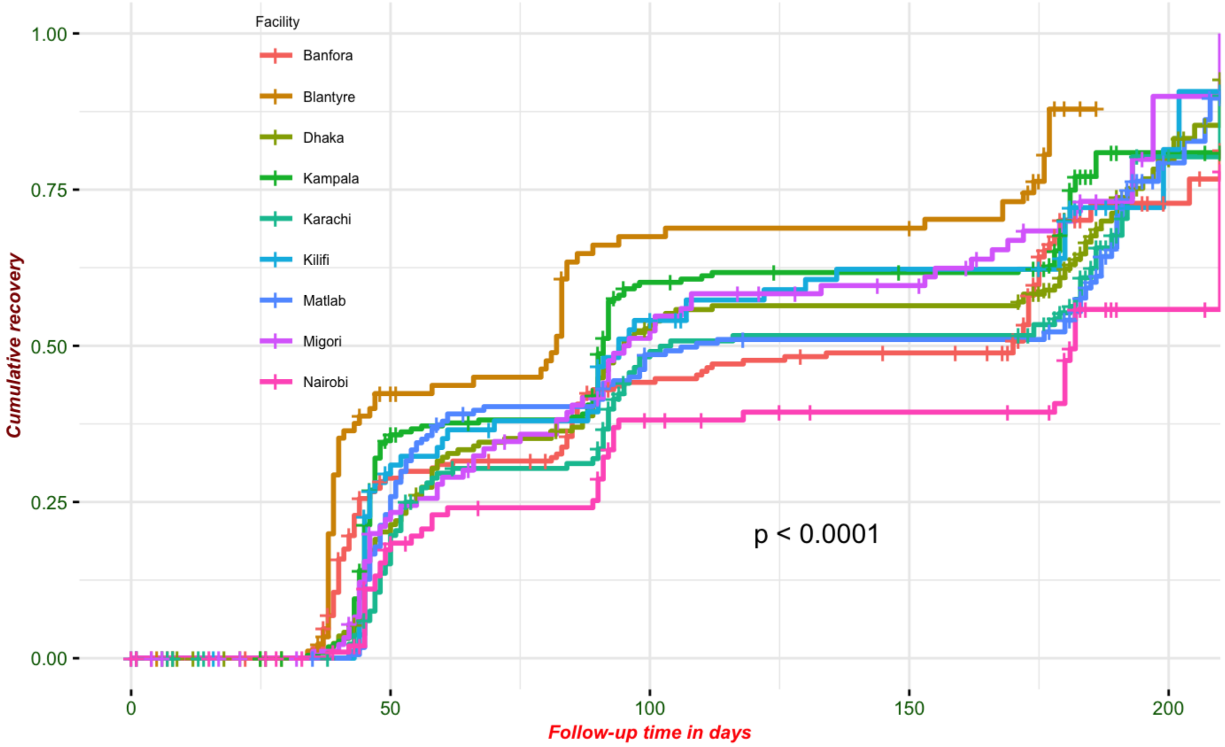

Figure S6. A Kaplan-Meier curve of recovery by country

Table S1: The association of food insecurity with dietary diversity

Characteristic	Overall, N = 1,286	Food secure, N = 460	Mild food insecurity, N = 307	Moderate food insecurity, N = 349	Severe food insecurity, N = 170	p-value[1]
**Sufficient dietary diversity, n (%)**	622 (48%)	274 (60%)	149 (49%)	141 (40%)	58 (34%)	<0.001

[1]Pearson's Chi-squared test

Table S2. Summary of Kaplan-Meir non-recovery probabilities

Time	At risk pop.	Recovered	Non-recovery probability	95% CI Lower	95% CI Upper
0	1286	0	1	*NA*	*NA*
15	1263	0	1	*NA*	*NA*
30	1249	0	1	*NA*	*NA*
45	1079	226	0.817	0.794	0.837
60	793	184	0.664	0.637	0.690
75	759	22	0.645	0.618	0.672
90	686	95	0.564	0.535	0.591
105	526	104	0.472	0.443	0.500
120	501	19	0.455	0.426	0.483
135	490	5	0.450	0.422	0.479
150	486	1	0.449	0.421	0.478
165	477	4	0.446	0.417	0.474
180	321	80	0.362	0.333	0.390

Median survival time was 96 days (95% CI: 92, 106).

Table S3. Summary of Kaplan-Meir non-recovery probabilities by dietary diversity

	Sufficient dietary diversity – YES Medians survival time – 98(95% CI:92,130)					Sufficient dietary diversity – No Medians survival time – 94(95% CI:91,1 07)				
Time	At risk pop.	Recovered	Non-recovery probability	95% CI Lower	95% CI Upper	At risk pop.	Recovered	Non-recovery probability	95% CI Lower	95% CI Upper
0	622	0	1.000	NA	NA	664	0	1	1	1
15	612	0	1.000	NA	NA	651	0	1	NA	NA
30	605	0	1.000	NA	NA	644	0	1	NA	NA
45	533	107	0.821	0.788	0.850	546	119	0.812	0.78	0.841
60	380	97	0.655	0.615	0.692	413	87	0.672	0.634	0.707
75	361	11	0.636	0.596	0.673	398	11	0.654	0.615	0.69
90	334	38	0.569	0.528	0.608	352	57	0.559	0.519	0.597
105	264	49	0.481	0.439	0.521	262	55	0.464	0.423	0.503
120	249	11	0.461	0.419	0.501	252	8	0.449	0.409	0.489
135	243	2	0.457	0.416	0.497	247	3	0.444	0.404	0.483
150	242	1	0.455	0.414	0.495	244	0	0.444	0.404	0.483
165	240	2	0.451	0.410	0.492	237	2	0.44	0.4	0.48
180	184	35	0.379	0.338	0.420	137	45	0.345	0.305	0.385

Table S4. Summary of Kaplan-Meir non-recovery probabilities by nutritional status

	MW (MUAC 11.5-12.5) Medians survival time – 81(95% CI:59,89)					SWK (MUAC<11.5 or edema) Medians survival time – 182(95% CI:180,186)				
Time	At risk pop.	Recovered	Non-recovery probability	95% CI Lower	95% CI Upper	At risk pop.	Recovered	Non-recovery probability	95% CI Lower	95% CI Upper
0	699	0	1	*NA*	*NA*	587	0	1	*NA*	*NA*
15	690	0	1	*NA*	*NA*	573	0	1	*NA*	*NA*
30	685	0	1	*NA*	*NA*	564	0	1	*NA*	*NA*
45	573	170	0.751	0.717	0.782	506	56	0.899	0.87	0.921
60	353	149	0.528	0.49	0.565	440	35	0.833	0.799	0.862
75	330	16	0.504	0.466	0.541	429	6	0.822	0.787	0.851
90	292	53	0.422	0.385	0.46	394	42	0.74	0.701	0.776
105	208	60	0.328	0.292	0.364	318	44	0.653	0.61	0.692
120	189	14	0.306	0.271	0.341	312	5	0.642	0.599	0.682
135	183	3	0.301	0.266	0.336	307	2	0.638	0.595	0.678
150	180	1	0.299	0.264	0.335	306	0	0.638	0.595	0.678
165	179	0	0.299	0.264	0.335	298	4	0.63	0.587	0.67
180	122	39	0.228	0.195	0.262	199	41	0.531	0.485	0.575

Table S5. Summary of Kaplan-Meir non-recovery probabilities by food insecurity

	Food secure<br>Medians survival time – 95(95% CI:91, 113)					Mild food insecurity<br>Medians survival time – 168(95% CI:96, 179)				
Time	At risk pop.	Recovered	Non-recovery probability	95% CI Lower	95% CI Upper	At risk pop.	Recovered	Non-recovery probability	95% CI Lower	95% CI Upper
0	460	0	1	NA	NA	307	0	1	1	1
15	450	0	1	NA	NA	304	0	1	NA	NA
30	447	0	1	NA	NA	296	0	1	NA	NA
45	403	72	0.837	0.800	0.869	242	62	0.786	0.735	0.829
60	287	77	0.660	0.614	0.703	188	35	0.664	0.606	0.715
75	274	7	0.644	0.597	0.687	182	4	0.649	0.591	0.701
90	249	35	0.562	0.514	0.607	168	15	0.594	0.535	0.649
105	191	42	0.462	0.414	0.508	141	18	0.527	0.467	0.584
120	184	4	0.452	0.404	0.498	133	7	0.501	0.441	0.558
135	179	3	0.444	0.397	0.491	132	0	0.501	0.441	0.558
150	179	0	0.444	0.397	0.491	131	0	0.501	0.441	0.558
165	175	1	0.442	0.394	0.488	130	0	0.501	0.441	0.558
180	121	25	0.372	0.325	0.419	88	20	0.414	0.354	0.473
	Moderate food insecurity<br>Medians survival time – 92(95% CI:90, 100)					Severe food insecurity<br>Medians survival time – 95(95% CI:90, 155)				
0	349	0	1	1	1	170	0	1	NA	NA
15	345	0	1	NA	NA	164	0	1	NA	NA
30	344	0	1	NA	NA	162	0	1	NA	NA
45	294	69	0.798	0.751	0.837	140	23	0.855	0.790	0.901
60	209	50	0.646	0.592	0.695	109	22	0.714	0.636	0.778
75	200	5	0.630	0.576	0.680	103	6	0.675	0.595	0.742
90	178	30	0.535	0.479	0.587	91	15	0.575	0.494	0.649
105	128	28	0.443	0.387	0.496	66	16	0.465	0.383	0.542
120	121	5	0.425	0.370	0.479	63	3	0.443	0.363	0.521
135	118	0	0.425	0.370	0.479	61	2	0.429	0.349	0.507
150	117	1	0.421	0.367	0.475	59	0	0.429	0.349	0.507
165	114	2	0.414	0.359	0.468	58	1	0.422	0.342	0.500
180	75	20	0.331	0.277	0.386	37	15	0.301	0.226	0.379

Table S6: Comparison of individuals with a follow-up MUAC and without a follow-up MUAC

		Overall	Have at least 1 MUAC	No follow-up MUAC
	n	1286	1180	106
Age				
	6-11 months	627 (49)	574 (49)	53 (50)
	12-17 months	436 (34)	400 (34)	36 (34)
	18-23 months	223 (17)	206 (17)	17 (16)
HIV-Positive		67 (5)	59 (5)	8 (8)
Wasting				
	Discharge to 45 days	47 (4)	2 (0)	45 (42)
	45-90 days	29 (2)	16 (1)	13 (12)
	90-180 days	546 (42)	531 (45)	15 (14)
	>180 days	664 (52)	631 (53)	33 (31)
Died		91 (7)	37 (3)	54 (51)
Baseline MUAC				
	SWK (MUAC<11.5)	748 (58)	675 (57)	73 (69)
	MW (MUAC11.5-12.5)	502 (39)	475 (40)	27 (25)
	No wasting (MUAC>12.5)	36 (3)	30 (3)	6 (6)
Stunting				
	Severe	469 (36)	429 (36)	40 (38)
	Moderate	376 (29)	358 (30)	18 (17)
	Mild	439 (34)	392 (33)	47 (44)
	Missing	2 (0)	1 (0)	1 (1)
Wasting				
	Severe	586 (46)	532 (45)	54 (51)
	Moderate	391 (30)	363 (31)	28 (26)
	Mild	307 (24)	284 (24)	23 (22)
	Missing	2 (0)	1 (0)	1 (1)
Died		91 (7)	37 (3)	54 (51)
Death likelihood at discharge				
	Almost certainly not	310 (24)	284 (24)	26 (25)
	Very unlikely	514 (40)	476 (40)	38 (36)
	Quite unlikely	324 (25)	298 (25)	26 (25)
	Unsure	107 (8)	93 (8)	14 (13)
	Quite likely	9 (1)	9 (1)	0 (0)
	Very likely	1 (0)	1 (0)	0 (0)
	Almost certainly	1 (0)	1 (0)	0 (0)
	NA	20 (2)	18 (2)	2 (2)
Lost to follow up status				
	Withdrew post-discharge	13 (1)	4 (0)	9 (8)
	Lost after discharge	9 (1)	4 (0)	5 (5)
	Did not withdraw/ltfu	1264 (98)	1172 (99)	92 (87)
Dietary diversity (sufficient)				
	No	664 (52)	613(52)	51 (48)

Yes	622 (48)	567 (48)	55 (52)
Food insecurity			
Mild	765 (59)	701 (59)	64 (60)
Moderate	349 (27)	326 (28)	23 (22)
Severe	172 (13)	153 (13)	19 (18)
Weight for length at admission (mean (SD))	-2.9 (1.3)	-2.9 (1.2)	-3.2 (1.3)
Weight for age at admission (mean (SD))	-3.5 (1.2)	-3.4 (1.2)	-3.6 (1.5)
Length for age at admission (mean (SD))	-2.6 (1.5)	-2.6 (1.5)	-2.5 (1.9)
MUAC at admission (mean (SD))	11.3 (1.0)	11.3 (1.0)	11.1 (1.1)
Weight for length at discharge (mean (SD))	-2.7 (1.2)	-2.6 (1.2)	-3.1 (1.4)
Weight for age at discharge (mean (SD))	-3.4 (1.2)	-3.3 (1.1)	-3.6 (1.5)
Length for age at discharge (mean (SD))	-2.7 (1.5)	-2.7 (1.5)	-2.6 (1.8)
MUAC at discharge (mean (SD))	11.4 (1.0)	11.4 (0.9)	11.1 (1.2)

Table S7: The distribution of specific food groups by breastfeeding status

	Breastfeeding		
Characteristic	No, N = 458[1]	Yes, N = 828[1]	p-value[2]
Grains	396 (86%)	703 (85%)	0.4
Legumes and nuts	216 (47%)	341 (41%)	0.038
Flesh foods	195 (43%)	350 (42%)	>0.9
Eggs	100 (22%)	271 (33%)	<0.001
Fruits and vegetables	241 (53%)	452 (55%)	0.5
Root and tubers	221 (48%)	396 (48%)	0.9
Milk & Dairy products	307 (67%)	447 (54%)	<0.001

2Pearson's Chi-squared test

Table S8: The relationship of wealth quintiles with breastfeeding status

Characteristic	No, N = 458[1]	Yes, N = 828[1]	p-value[2]
**Wealth quintiles**			0.2
Least poor	73 (16%)	167 (20%)	
Fourth	111 (24%)	169 (20%)	
Middle	102 (22%)	167 (20%)	
Second	88 (19%)	164 (20%)	
Poorest	84 (18%)	161 (19%)	

[1]n (%)

[2]Pearson's Chi-squared test

Table S9: The distribution of specific food groups, food insecurity, dietary diversity, and study setting by fruits and vegetables

Characteristic	0, N = 593[1]	1, N = 693[1]
**Breast feeding**	376 (45%)	452 (55%)
**Grains**	458 (42%)	641 (58%)
**Legumes and nuts**	138 (25%)	419 (75%)
**Flesh foods**	146 (27%)	399 (73%)
**Eggs**	51 (14%)	320 (86%)
**Root and tubers**	133 (22%)	484 (78%)
**Milk & Diary products**	302 (40%)	452 (60%)
**Food groups consumed**		
1	73 (100%)	0 (0%)
2	237 (89%)	29 (11%)
3	150 (69%)	68 (31%)
4	76 (46%)	91 (54%)
5	42 (25%)	127 (75%)
6	12 (7.6%)	145 (92%)
7	3 (2.2%)	135 (98%)
8	0 (0%)	98 (100%)
**Sufficient dietary diversity**	188 (30%)	434 (70%)
**Nutritional status at discharge**		
MAM (MUAC 11.5-12.5)	298 (43%)	401 (57%)
SAM (MUAC<11.5 or edema)	295 (50%)	292 (50%)
**Facility**		
Banfora	166 (85%)	30 (15%)
Blantyre	39 (42%)	53 (58%)
Dhaka	96 (55%)	78 (45%)
Kampala	121 (54%)	103 (46%)
Karachi	75 (52%)	68 (48%)
Kilifi	40 (52%)	37 (48%)
Matlab	11 (6.2%)	165 (94%)
Migori	40 (42%)	56 (58%)
Nairobi	5 (4.6%)	103 (95%)

[1] n (%)

RESULTS BY STUDY SITE

Table S10: The distribution of food security and dietary diversity by study site

Characteristic	Banfora, N = 196[1]	Blantyre, N = 92[1]	Dhaka, N = 174[1]	Kampala, N = 224[1]	Karachi, N = 143[1]	Kilifi, N = 77[1]	Matlab, N = 176[1]	Migori, N = 96[1]	Nairobi, N = 108[1]
**Food security status**									
Food secure	77 (39%)	19 (21%)	90 (52%)	38 (17%)	31 (22%)	18 (23%)	130 (74%)	37 (39%)	20 (19%)
Mild food insecurity	95 (48%)	11 (12%)	29 (17%)	55 (25%)	25 (17%)	20 (26%)	44 (25%)	4 (4.2%)	24 (22%)
Moderate food insecurity	24 (12%)	33 (36%)	33 (19%)	99 (44%)	56 (39%)	20 (26%)	2 (1.1%)	29 (30%)	53 (49%)
Severe food insecurity	0 (0%)	29 (32%)	22 (13%)	32 (14%)	31 (22%)	19 (25%)	0 (0%)	26 (27%)	11 (10%)
**Sufficient dietary diversity**	56 (29%)	44 (48%)	100 (57%)	70 (31%)	54 (38%)	27 (35%)	159 (90%)	31 (32%)	81 (75%)

[1] n (%)

Table S11: The distribution food types by study site

Characteristic	Banfora, N = 196[1]	Blantyre, N = 92[1]	Dhaka, N = 174[1]	Kampala, N = 224[1]	Karachi, N = 143[1]	Kilifi, N = 77[1]	Matlab, N = 176[1]	Migori, N = 96[1]	Nairobi, N = 108[1]
**Breast Milk**	161 (82%)	53 (58%)	127 (73%)	78 (35%)	82 (57%)	42 (55%)	162 (92%)	41 (43%)	82 (76%)
**Milk Products**	50 (26%)	17 (18%)	102 (59%)	133 (59%)	110 (77%)	30 (39%)	143 (81%)	72 (75%)	97 (90%)
**Egg**	10 (5.1%)	11 (12%)	33 (19%)	51 (23%)	38 (27%)	2 (2.6%)	150 (85%)	26 (27%)	50 (46%)
**Flesh Foods**	57 (29%)	34 (37%)	35 (20%)	155 (69%)	30 (21%)	9 (12%)	140 (80%)	34 (35%)	51 (47%)
**Grains**	168 (86%)	91 (99%)	153 (88%)	188 (84%)	68 (48%)	76 (99%)	167 (95%)	85 (89%)	103 (95%)
**Root and tubers**	13 (6.6%)	14 (15%)	81 (47%)	173 (77%)	37 (26%)	11 (14%)	153 (87%)	35 (36%)	100 (93%)
**Legumes and Nuts**	23 (12%)	53 (58%)	76 (44%)	153 (68%)	22 (15%)	18 (23%)	103 (59%)	36 (38%)	73 (68%)
**Fruits and Vegetables**	30 (15%)	53 (58%)	78 (45%)	103 (46%)	68 (48%)	37 (48%)	165 (94%)	56 (58%)	103 (95%)

[1]n (%)

Table S12: Proportion of recovery by facility

Characteristic	Overall, N = 1,286	0, N = 461	Recovered, N = 825	p-value[1]
**Facility, n (%)**				<0.001
Banfora	196 (15%)	78 (40%)	118 (60%)	
Blantyre	92 (7.2%)	26 (28%)	66 (72%)	
Dhaka	174 (14%)	50 (29%)	124 (71%)	
Kampala	224 (17%)	72 (32%)	152 (68%)	
Karachi	143 (11%)	58 (41%)	85 (59%)	
Kilifi	77 (6.0%)	26 (34%)	51 (66%)	
Matlab	176 (14%)	58 (33%)	118 (67%)	
Migori	96 (7.5%)	32 (33%)	64 (67%)	
Nairobi	108 (8.4%)	61 (56%)	47 (44%)	

[1]Pearson's Chi-squared test

Table S13: Kaplan Meier test result comparing time to recovery acrsso stusy sites

Facility	N	Observed	Expected
Banfora	196	118	116.1
Blantyre	92	66	34.2
Dhaka	174	124	128.1
Kampala	224	152	127.8
Karachi	143	85	102.6
Kilifi	77	51	47.6
Matlab	176	118	136.7
Migori	96	64	58.2
Nairobi	108	47	73.7
Chisq= 52.4 on 8 degrees of freedom, p= 1e-08			

Table S14: Crude HR for the association of study site with recovery

Facility	HR	2.50%	97.50%
Banfora	0.84	0.66	1.07
Blantyre	1.62	1.22	2.17
Dhaka	0.8	0.63	1.02
Karachi	0.68	0.52	0.89
Kilifi	0.9	0.65	1.23
Matlab	0.71	0.56	0.91
Migori	0.92	0.68	1.23
Nairobi	0.53	0.38	0.73
Ref: Kampala – higher sample size			

Table S15: The interaction of age with specific food groups, dietary diversity, and food insecurity

Group	Characteristic	HR[1]	95% CI[1]	p-value
Breastfeeding	**Age**			
	<12 Months	—	—	
	>= 12 Months	1.58	1.25, 2.00	<0.001
	**Breast Milk**	1.01	0.81, 1.26	>0.9
	**Age * Breast Milk**			
	>= 12 Months * Breast Milk	0.78	0.58, 1.04	0.088
Milk and dairy products	**Age**			
	<12 Months	—	—	
	>= 12 Months	1.46	1.17, 1.83	<0.001
	**Milk and dairy products**	1.06	0.85, 1.32	0.6
	**Age * Milk and dairy products**			
	>= 12 Months * Milk and diary products	0.89	0.67, 1.19	0.4
Flesh foods	**Age**			
	<12 Months	—	—	
	>= 12 Months	1.32	1.09, 1.59	0.004
	**Flesh foods**	1.09	0.88, 1.34	0.4
	**Age * Flesh foods**			
	>= 12 Months * Flesh foods	1.04	0.78, 1.37	0.8
Grains	**Age**			
	<12 Months	—	—	
	>= 12 Months	1.75	1.19, 2.57	0.004
	**Grains**	1.36	1.03, 1.81	0.03
	**Age * Grains**			
	>= 12 Months * Grains	0.74	0.49, 1.11	0.14
Legumes and nuts	**Age**			
	<12 Months	—	—	
	>= 12 Months	1.26	1.04, 1.52	0.016
	**Legumes and nuts**	1.12	0.91, 1.37	0.3
	**Age * Legumes and nuts**			
	>= 12 Months * Legumes and nuts	1.15	0.87, 1.53	0.3
Egg	**Age**			
	<12 Months	—	—	
	>= 12 Months	1.44	1.22, 1.70	<0.001
	**Egg**	0.98	0.78, 1.22	0.8
	**Age * Egg**			
	>= 12 Months * Egg	0.83	0.61, 1.13	0.2
Fruits and vegetables	**Age**			
	<12 Months	—	—	
	>= 12 Months	1.19	0.97, 1.47	0.09

	**Fruits and vegetables**	0.84	0.68, 1.03	0.088
	**Age * Fruits and vegetables**			
	>= 12 Months * Fruits and vegetables	1.28	0.97, 1.69	0.086
Food insecurity	**Age**			
	<12 Months	—	—	
	>= 12 Months	1.36	1.08, 1.71	0.01
	**Food insecurity**			
	Food secure	—	—	
	Mild food insecurity	0.88	0.68, 1.16	0.4
	Moderate food insecurity	1.12	0.87, 1.45	0.4
	Severe food insecurity	1.15	0.82, 1.62	0.4
	**Age * Food insecurity**			
	>= 12 Months * Mild food insecurity	1.08	0.75, 1.57	0.7
	>= 12 Months * Moderate food insecurity	0.98	0.69, 1.39	>0.9
	>= 12 Months * Severe food insecurity	0.89	0.57, 1.38	0.6
Dietary diversity	**Age**			
	<12 Months	—	—	
	>= 12 Months	1.45	1.18, 1.77	<0.001
	**Sufficient dietary diversity**			
	No	—	—	
	Yes	1.07	0.87, 1.32	0.5
	**Age * Sufficient dietary diversity**			
	>= 12 Months * Yes	0.89	0.67, 1.18	0.4

[1]HR = Hazard Ratio, CI = Confidence Interval
